# A Different Facet of p53 Function: Regulation of Immunity and Inflammation During Tumor Development

**DOI:** 10.3389/fcell.2021.762651

**Published:** 2021-10-18

**Authors:** Di Shi, Peng Jiang

**Affiliations:** ^1^School of Life Sciences, Tsinghua University, Beijing, China; ^2^Tsinghua-Peking Center for Life Sciences, Beijing, China

**Keywords:** p53, immune, inflammation, tumor microenvironment (TME), innate and adaptive immune response

## Abstract

As a key transcription factor, the evolutionarily conserved tumor suppressor p53 (encoded by *TP*53) plays a central role in response to various cellular stresses. A variety of biological processes are regulated by p53 such as cell cycle arrest, apoptosis, senescence and metabolism. Besides these well-known roles of p53, accumulating evidence show that p53 also regulates innate immune and adaptive immune responses. p53 influences the innate immune system by secreted factors that modulate macrophage function to suppress tumourigenesis. Dysfunction of p53 in cancer affects the activity and recruitment of T and myeloid cells, resulting in immune evasion. p53 can also activate key regulators in immune signaling pathways which support or impede tumor development. Hence, it seems that the tumor suppressor p53 exerts its tumor suppressive effect to a considerable extent by modulating the immune response. In this review, we concisely discuss the emerging connections between p53 and immune responses, and their impact on tumor progression. Understanding the role of p53 in regulation of immunity will help to developing more effective anti-tumor immunotherapies for patients with *TP53* mutation or depletion.

## Introduction

As an intensively studied protein, the fame of p53 mainly stemming from its role as a tumor suppressor which is activated when responding to stress signals such as genotoxic damage, or nutrient deprivation (Lowe et al., 2004; Vousden and Lane, 2007; Levine and Oren, 2009). Mutations of p53 always accompanied dysregulation of metabolism, migration, and invasion, all of which ultimately result in the development of clinical tumors and an ever more aggressive malignancy (Hanahan and Weinberg, 2011; Jiang et al., 2013; Schwitalla et al., 2013; Labuschagne et al., 2018). Cancer cells can be recognized and destructed by innate and adaptive immune effector cells, a process that is known as cancer immunosurveillance (Zitvogel et al., 2006). In recent years, various studies have indicated that p53 can also control tumor-immune system crosstalk (Watanabe et al., 2014; Guo and Cui, 2015; Blagih et al., 2020b). p53 loss in tumors provokes an altered myeloid and T cell responses. Specifically, p53 loss increases myeloid infiltration through enhanced secretion of cytokines (Blagih et al., 2020b). Morever, dysfunction of p53 under certain circumstance reprograms the components of tumor microenvironment (TME), leading to an altered immunologic milieu which exacerbates tumor progression. Here, we review the latest understanding of p53 in regulating the immune response during tumor development.

## p53 Regulation of Inflammation

Besides the capability of governing cellular homeostasis to curb tumourigenesis, accumulating observations suggest that p53 also plays the role in inflammatory reactions (Gudkov et al., 2011; Cooks et al., 2014). Chronic inflammation creates a potential cancer-promoting condition (Karin, 2006; Mantovani et al., 2008). In inflamed tissues, cytokines or inflammatory mediators can activate several transcription factors such as NF-κB and Signal Transducer and Activators of Transcription 3 (STAT3) which are critical in promoting cancer initiation. The activation of NF-κB and STAT pathways results in the enrichment of ROS in TME which ultimately prompts chronic inflammation (Trinchieri, 2012). Accumulating evidence strongly indicate that p53 dysfunction in tumors can enhance chronic inflammation and then promote tumor progression. Below, we discuss the role of p53 in inflammation.

### NF-κB and p53

Chronic inflammation enhances the risk of cancer. As the crucial transcription factor, NF-κB is constitutively activated in most tumors. p53 and NF-κB pathways play crucial roles in response to various stresses and the NF-κB activity usually shows an antagonistic relationship with that of p53 (Kawauchi et al., 2008a; Ak and Levine, 2010; Gudkov et al., 2011). In contrast to p53 whose canonical role is growingly restrictive, NF-κB vastly promotes cell survival and inflammation. NF-κB and p53 have an extensive crosstalk in numerous cancers. Specifically, chronically inflamed and malignant tissues are always accompanied by constitutive activation of NF-κB where the p53 function is repressed by persistent infections or tissue irritating factors (Webster and Perkins, 1999; Schneider et al., 2010; Son et al., 2012; Natarajan et al., 2014). Mice with intestinal epithelial cell (IEC)-specific p53 deficiency do not initiate intestinal tumorigenesis, but significantly enhance carcinogen-induced tumourigenesis by promoting the establishment of an NF-κB-dependent inflammatory microenvironment that increases intestinal permeability and further invasion and metastasis (Schwitalla et al., 2013). Moreover, activated p53 acts as a suppressor directly suppressing the transcriptional activity of NF-κB, and aberrant inflammation can enhance tumor development when p53 is lost (Kawauchi et al., 2008a, b; Son et al., 2012; Gudkov and Komarova, 2016; Uehara and Tanaka, 2018).

Intriguingly, the reciprocal activation of p53 and NF-κB has been also found in certain cases (Lowe et al., 2014). It has been reported that p53 and NF-κB co-regulate the induction of pro-inflammatory genes, such as IL-6 and CXCL1, in human macrophages to drive the induction of pro-inflammatory cytokines (Lowe et al., 2014). Moreover, the activation of NF-κB promotes the secretion of numerous inflammatory cytokines and chemokines in senescent cells with highly activated p53 (Rodier and Campisi, 2011; Davalos et al., 2013).

As the most frequently genetic alterations in cancer, p53 mutations exist in over half of human cancers. However, many p53 mutants (mutp53) gain new activities to augment pro-inflammatory and survival properties, termed gain-of-function (GOF). Several studies have shown that GOF mutp53 can activate some of the NF-κB target genes (Cooks et al., 2013; Di Minin et al., 2014; Rahnamoun et al., 2017). For example, Cooks et al. (2013) demonstrated that mutp53 prolong NF-κB activation, leading to a significant proinflammatory activity and promoting colitis-associated colorectal cancer in mouse model. Di Minin et al. (2014) reported that mutp53 in cancer cells reprogram NF-κB and JNK activation in response to TNFα through the binding and interfering the tumor suppressor RasGAP Disabled 2 Interacting Protein (DAB2IP) in the cytoplasm. Mutp53 can also interact with NF-κB directly, enhancing RNA polymerase II recruitment in response to chronic TNF signaling which shapes the enhancer landscape and oncogenic gene expression (Rahnamoun et al., 2017). Therefore, inhibition of NF-κB to restore wild-type (WT) p53 function or reactivation of WT p53 in the context of mutp53 would be a very attractive target for cancer therapy.

### Small Molecule Modulators Simultaneously Activate p53 and Inhibit NF-κB

As mentioned above, killing strategies that directly target the p53 and NF-κB pathways can be utilized to improve cancer therapy (Cheok et al., 2011; Khoo et al., 2014; Muller and Vousden, 2014). Several moleculers targeting both pathways have been indentified and some of which are already in clinical trials. For example, anti-malaria drug quinacrine was identified to have the ability to kill cancer cells by simultaneously inhibiting NF-κB and activating p53 (Gurova et al., 2005). Quinine and other aminoacridine derivatives mimic DNA damage, are non-genotoxic, and have good therapeutic potential for cancer in mouse xenograft models. This is noteworthy because anticancer drugs such as cisplatin induce p53 by forming covalent DNA adducts. r-Roscovitine, another small molecule, targets multiple signaling pathways simultaneously and prevents tumor growth. It activates p53 while blocking NF-κB activity and has shown its anticancer properties in phase II clinical trials (Lu et al., 2001; Dey et al., 2008). Interestingly, r-Roscovitine was originally developed as a cell cyclin-dependent kinase (CDK) inhibitor, which was shown to inhibit MDM2 expression and stabilize p53 (Lu et al., 2001). r-Roscovitine downregulates NF-κB activation in response to TNF-α and IL-1 by inhibiting IκB kinase (IKK) activity. It also inhibits the phosphorylation of p65 at Ser536 *via* IKK, which is required for nuclear localization. At the transcriptional level, r-Roscovitine inhibits the transcription of NF-κB-regulated genes such as MCP-1, ICAM-1, COX2, FLIP, and IL-8 (Dey et al., 2008). Nutlin is the first Mdm2 antagonist reported to inhibit the p53-Mdm2 interaction and was shown to inhibit tumor growth in mouse models (Vassilev et al., 2004; Tovar et al., 2006). It was shown that Nutlin also strongly inhibits the protein expression of NF-κB target genes ICAM-1 and MCP-1, depending on p53 status (Dey et al., 2007). Clearly, more research is needed to better understand the mechanisms behind these drugs and to find more small molecules with higher specificity to activate p53 and inhibit NF-κB.

### p53 and Signal Transducer and Activators of Transcription Pathways

Signal transducer and activators of transcription family is a group of transcription factors that regulate cytokine-dependent inflammation and immunity (Grivennikov et al., 2010). Constitutively activated STATs, especially STAT3, induce and maintain a protumourigenic inflammatory microenvironment to stimulate the initiate and survival of malignant cells (Catlett-Falcone et al., 1999; Mantovani et al., 2008; Grivennikov et al., 2009). p53 regulates inflammation response through STAT3 that is activated by inflammatory cytokine IL-6. And, p53 loss in pancreatic cancer results in activated STAT3 phosphorylation, which is initiated by IL-6 (Wormann et al., 2016). Like NF-κB, STAT3 binds to the p53 promoter directly to inhibit p53 transcription, limiting its canonical tumor suppressor function. Blocking STAT3 activates expression of p53, leading to p53-dependent tumor cell apoptosis (Niu et al., 2005). It has been shown that tumor cells dependent on long-term STAT3 signaling are more sensitive to STAT3 inhibitors than normal cells (Yu and Jove, 2004). Thus, STAT3 proteins can be targeted as novel cancer therapeutics, and more effective and selective STAT inhibitors can be expected to be developed in the future.

Besides the reciprocal relationship of STAT3 and p53, it has also been reported that inactivation of p53 in macrophages results in elevated levels of total and phosphorylated STAT1, thereby increases the production of proinflammatory cytokines (Zheng et al., 2005). Furthermore, p53 stimulates Treg cell differentiation *via* direct interaction with STAT5 (Park et al., 2013). Therefore, it is likely that p53 can balance the activity of various STAT pathways to impact host immune response.

## Cellular Constituents of the Tumor Microenvironment

Emerging studies suggest that tumor cell growth and invasion are markedly affected by tumor microenvironment (TME) (Kerkar and Restifo, 2012; Swartz et al., 2012). The TME contains not only cells but also signaling molecules, extracellular matrix, and mechanical cues. The immunological landscape of TME is shaped by all these cellular and molecular components that support neoplastic transformation, protects the cancer cells from host immunity, and provides niches for metastasis. Besides the cell-autonomous effects of p53, emerging evidence show that p53 can also have effects on neighboring cells, i.e., non-cell-autonomous activities of p53 (Bar et al., 2010; Lujambio et al., 2013). Thus, better understanding the function of p53 in TME may be potentially used to tailor personalized therapies for patients with tumors bearing p53 mutations.

### Cancer-Associated Fibroblasts

**FIGURE 1:**
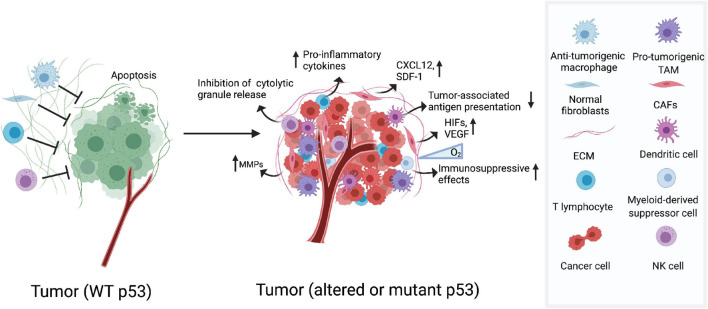
Dysfunction of p53 shapes its immunogenic niche. The TME contains cellular and molecular components that shape immunological landscape of growing tumors. Altered or mutant p53 functions in CAFs increases expression of chemokines and cytokines, which drives tumor invasion and malignancy. In hypoxic tumor environments, GOF mutp53 cooperate with HIF-1 promoting expression of a subset of protumourigenic genes which participate in the rearrangement of ECM. In myeloid cells, p53 deficiency helps to accelerate tumourigenesis. Deletion of p53 in T cells increases the expression of pro-inflammatory cytokines which could help to enhance tumor progression.

In the TME, cancer-associated fibroblasts (CAFs) play an important role in modulating tumor progression and metastasis (Ohlund et al., 2014; Kalluri, 2016). In CAFs of highly inflamed cancers, p53 mutations are frequently detected (Patocs et al., 2007). The tumor inflammatory milieu can be affected by altered p53 status in CAFs which is accompanied by an increased rate of tumor metastasis and worse prognosis. Mechanically, p53 dysfunction in CAFs can promote tumor invasion and malignancy through upregulation of chemokines and cytokines, including CXCL12 and SDF-1 (Figure 1; Moskovits et al., 2006; Addadi et al., 2010). Surprisingly, Arandkar et al. (2018) found that non-mutated CAF p53 is functionally distinct from normal fibroblast p53. p53 in lung-derived CAFs is usually hypophosphorylated and is able to modify the transcriptional program, affect the CAF secretome, and promot cancer cell migration and invasion. Overall, tumor progression may require functionally altered p53 in CAFs, and it can be speculated that agents capable of “re-educating” p53 in cancer-associated stromal cells may be able to provide clues for cancer therapy (Arandkar et al., 2018).

### Extracellular Matrix Remodeling

One of the most important components in TME is the extracellular matrix (ECM), which are comprised of various macromolecules that regulate cellular functions in tumors. Tumor cells manipulate the arrangement and orientation of ECM to enhance tumor progression and create a positive tumourigenic feedback loop (Cox and Erler, 2011). Previous studies have demonstrated that p53 expression and nuclear localization are modulated by ECM signals (Li et al., 2003). In recent years, the role of p53 in regulating ECM has been verified especially in hypoxic contexts (Petrova et al., 2018). In hypoxic tumor environments, the activation of transcription factors hypoxia inducible factors (HIF) results in the expression of pro-angiogenic factors such as vascular endothelial growth factor (VEGF), which directly participate in the rearrangement of ECM. It has recently been reported that the formation of HIF-1/GOF mutp53 complex in hypoxic cancer cells promotes the transcription of protumourigenic genes and codifys the components of ECM (Figure 1; Amelio et al., 2018). p53 can also negatively regulate extracellular matrix metalloproteinase inducer (EMMPRIN), a transmembrane glycoprotein known to promote metastasis and invasion of tumor by enhancing the production of several matrix metalloproteinases (MMPs) (Figure 1; Zhu et al., 2009). All these findings underscore the importance that restoring the function of p53 in the ECM may help in the development of anti-cancer therapies.

### Immune Cells

Immune cells are important cellular compartments in TME that are heterogeneous across tumor types and are associated with cancer progression and prognosis (Angell and Galon, 2013; Sun et al., 2015; Mlecnik et al., 2016a, b). Productive antitumour immunity largely relies on the tumor-reactive T cells. However, the cytotoxicity of T cells are frequently frustrated in the TME, where the cross-talk between MDSC, macrophages, DC and Treg amplifies the anti-tumor immune effects (Ostrand-Rosenberg et al., 2012).

The function of immune cells can also be regulated by p53. Previous studies have reported that p53^–/–^ mice show more susceptibility to inflammation and auto-immunity which favors tumor establishment and progression (Okuda et al., 2003; Zheng et al., 2005; Guo et al., 2017). And the function of p53 in various immune cells has also been dissected. For instance, p53 deficiency in myeloid lineage accelerates tumourigenesis in an intestinal cancer model, and activation of p53 attenuates the inflammatory response and resists tumor development (Figure 1; Guo et al., 2013; He et al., 2015). Furthermore, deficiency of p53 in T cells spontaneously develops inflammatory lesions and autoimmunity, which may help promote tumor development (Zhang et al., 2011).

However, p53 also has a role in regulating the polarization of CD4^+^ T cells by enhancing the transcription of Foxp3, a master regulator of Tregs, which predicts that the loss of this role of p53 could enhance antitumour immunity (Kawashima et al., 2013). Moreover, deletion of p53 in cytolytic T cells exhibits enhanced glycolytic commitment and reduces murine melanoma (Banerjee et al., 2016). The concept that p53 deletion in T cells enhances antitumour immunity is interesting. However, it may be influenced by other stromal compartments, as p53-deficient mice have substantially faster subcutaneous tumor growth and more regulatory T cells compared to wild-type controls (Guo et al., 2013).

More recently, studies from Dr. Weiping Zou’s team reveal that targeting p53–MDM2 interactions augments MDM2 in T cells, thereby stabilizing STAT5 and improving T cell-mediated anti-tumor immunity. Interestingly, these effects are independent of the p53 status of the tumor. Therefore, targeting this pathway could be explored to develop and select additional MDM2-targeted drugs independent of tumor p53 status (Zhou et al., 2021).

Together, these results highlight the important role of p53 in maintaining appropriate TME to suppress tumourigenesis and the potential development of new therapeutic approaches by targeting the p53 pathway.

Compelling evidence suggests that effective cancer therapy requires a multifaceted and integrated approach that not only exposes the tumor but also induces strong anti-tumor immunity. However, current approaches have focused on activating or restoring p53 function in cancer cells. As mentioned above, activation of p53 in TME also affects the immune response. Furthermore, local activation of the p53 pathway rather than overall activation may be sufficient to cause tumor death. Therefore, activation of p53 in TME is an exciting strategy for improving antitumour therapy in the future.

## p53 Functions in Innate and Adaptive Immunity

### The Role of p53 in Innate Immunity

As the first line of defense to detect invaders, innate immune cells are engaged in immediate short-term immune operations upon detection of pathogenic threats to attack and engulf the outsider without establishing immunological memory. The activation of innate immunity is initiated by the stimulation of cell-surface or intracellular pattern recognition receptors (PRRs), including retinoic-acid- inducible gene I (RIG-I)-like receptors (RLRs), stimulator of IFN genes (STING) protein, and Toll-like receptors (TLRs) (Kawai and Akira, 2010; Trinchieri, 2010; Burdette et al., 2011). The role of p53 in antiviral response has been well reviewed. Here, we discuss how p53 functions in innate immunosurveillance of tumor cells.

The TLRs are membrane glycoproteins and previous studies reveal that p53 transcriptionally regulate several TLRs, constituting a crucial bridge between cellular stresses and TLR-induced innate immune response (Taura et al., 2008; Menendez et al., 2011). Notably, TLR4 has been reported to possess dichotomous role during breast cancer growth, based on the status of p53. TLR4 activation in wtp53 cancer cells leads to the secretion of anti-inflammatory cytokines into microenvironment, resulting in the induction of p21 and cell growth arrest. By contrast, TLR4 activation in mutp53 cells increases secretion of progrowth cytokines such as CXCL1 and CD154. Furthermore, the influence of p53 status on TLR4 activity may extend across cancer types, suggesting that the connection between TLR4 and p53 may provide a therapeutic clue for specifically targeting mutp53 tumors (Haricharan and Brown, 2015).

**FIGURE 2:**
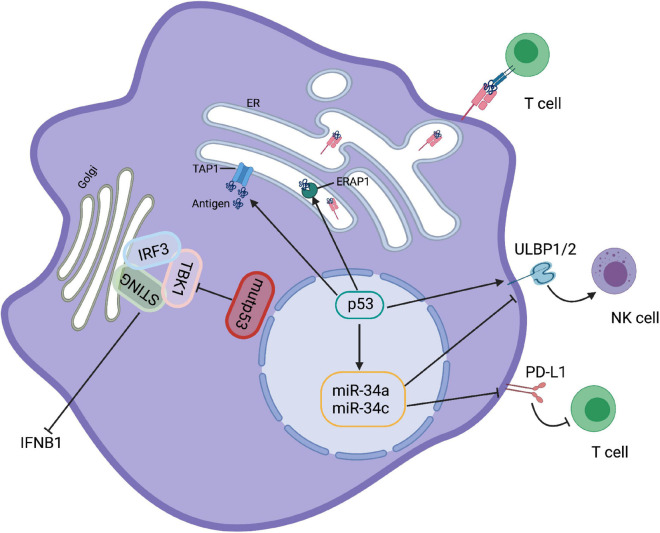
p53 regulates immune responses. Mutp53 binds to TBK1 preventing the formation of STING-TBK1-IRF3 trimeric complexes and rendering immune evasion. p53 upregulation ULBP1 and ULBP2, the NKG2G ligands, to enhance NK cell-mediate cytotoxicity. However, activation of p53 in melanoma cells increases the expression level of miR34, a ULBP2 inhibitor that reduces the recognition of tumor cells by NK cells. Moreover, p53 and miR-34a can cooperate to regulate tumor immune evasion *via* PD-L1. In addition, activation of p53 is able to promote the transport and expression of MHC-I by upregulating TAP1 and ERAP1.

The cGAS-STING pathway also plays essential role in anti-tumor immunity *in vivo via* up-regulation of type I IFNs (Ablasser and Chen, 2019). More recently, Ghosh et al. (2021) reported that GOF activity of mutp53 can antagonize the STING/TBK1/IRF3 pathway. Mutp53, but not wtp53, binds to TANK-binding protein kinase 1 (TBK1), preventing the formation of the STING-TBK1-IRF3 trimeric complex, which is required for cytokine production and ultimately leads to the onset of immune evasion (Figure 2). This finding may provide a key clue to therapeutic approaches aimed at restoring TBK1 function to reactivate immunosurveillance in mutp53-expressing tumors.

Besides myeloid cells, NKG2D-mediated NK cells are also regulated by p53. Restoration of p53 upregulates cell surface expression of ULBP1 and ULBP2 (the NKG2D ligands) that enhance NK cell-mediated cytotoxicity (Textor et al., 2011). However, activation of p53 by Nutlin-3a reduces the expression of ULBP2 in melanoma cells due to the induction of miR-34a/c (Heinemann et al., 2012; Figure 2). Thus, it appears that the effect of p53 activation on innate immune regulation is governed by the conditions of its induction.

### The Role of p53 in Adaptive Immunity

The development of effective immunotherapies for oncology patients is now becoming a clinical reality. Notably, the interaction between T cells and DCs is developing as one of the key targets for immunotherapy. As an important sensor to activate adaptive immune responses, p53-mediated activation of innate immune cells, particularly DC, is expected to promote adaptive immunity. Although the direct effect of p53 on the function of DCs has not been clarified, many results suggest that p53 activation is necessary for DC function. Treatment with Nutlin 3, an MDM2 inhibitor that activates wild-type p53, has been reported to increase the ability of DCs to stimulate T-cell proliferation, suggesting that p53 is involved in the activation of DCs (Gasparini et al., 2012). It has also been shown that the induction of p53 promotes peptide processing and MHC-I expression on the cell surface (Figure 2; Zhu et al., 1999; Wang et al., 2013). Therefore, it can be speculated that the enhancement of DC function by p53 may further improve the induction of cytotoxic CD8^+^ T cells, and the direct role of p53 in DC antigen presentation requires further exploration.

Cancer cells normally upregulate immune checkpoint molecules such as programmed cell death 1 ligand 1 (PD-L1) and cytotoxic T lymphocyte antigen 4 (CTLA4), which are important for T cell tolerance to evade immune attack (Le Mercier et al., 2015; Sharma and Allison, 2015; Baumeister et al., 2016). Links between p53 and immune checkpoints have recently been uncovered. IFN-γ-induced upregulation of PD-L1 expression in melanoma is dependent on p53 (Thiem et al., 2019). Moreover, a number of microRNAs (miRs), which are targets for p53, also play an important role in adaptive and innate immunity. For example, as a transcriptional target of p53, miR-34a inhibits the expression of PD-L1, and dysfunction of p53 increases PD-L1 expression, thereby suppressing T-cell function (Figure 2). This result indicates that p53 and miR-34a cooperate to regulate tumor immune evasion *via* PD-L1 (Cortez et al., 2016). Consistent with this, tumor cells carrying p53 dysfunction are usually accompanied with increased expression of PD-L1, which may help to identify patients who respond to immune checkpoint inhibitors against PD-L1 (Cha et al., 2016; Cortez et al., 2016; Biton et al., 2018; Blagih et al., 2020a).

## p53 and Dead Cell Clearance

During the resolution of injury and infection, normal cell turnover and clearance is an important process in preventing autoimmunity and triggering immune recognition of antigens by dying cells (Green et al., 2009). Failure to sustain efficient clearance is the key contributor to foster disease such as cancer and chronic inflammatory (Elliott and Ravichandran, 2010; Nagata et al., 2010; Fuchs and Steller, 2011; Arandjelovic and Ravichandran, 2015). In normal immune system, phagocytosis of dying cells can induce some degree of immune tolerance to prevent self-antigen recognition. p53 is well-documented as an important regulator of apoptosis, and the role of p53 involved in post-apoptosis has been recently identified. The immune checkpoint regulator DD1α has been reported to be a direct transcriptional target of p53. p53-induced expression of DD1α enhances clearance of apoptotic cells by promoting phagocytosis of macrophages, suggesting that p53 provides protection against inflammatory diseases caused by apoptotic cell accumulation (Yoon et al., 2015). Interactions between macrophage DD1α and T cell DD1α were also observed, making them susceptible to immunosuppression (Zitvogel and Kroemer, 2015). Therefore, this association warrants further preclinical characterization as a potential therapeutic target.

## Potential of p53 in Immunotherapy

As mentioned above, the regulation of p53 in the tumor immune response exhibits a yin-yang balance. On the one hand, p53 counteracts pro-inflammatory factors, such as NF-κB and STAT3, to promote tissue homeostasis and avoid excessive immune responses. On the other hand, p53 contributes to the recognition of non-self antigens and thus activates anti-tumor immunity through multiple pathways. All these p53 features will allow us to develop more effective tumor therapies in combination with current immunotherapies.

### Mutant p53 as a Tumor Antigen

Cancer cells are always accompanied by unstable genetic changes and produce new antigens that distinguish cancer cells from normal cells. The accumulation of p53 hotspot mutations in cancer has been considered as immunologically active neoantigens for immunotherapy. However, progress in this field has been limited by the lack of efficiency of recognition of mutant p53 antigens in cells (Yen et al., 2000; Nijman et al., 2005; Lane et al., 2011). A recent clinical trial in metastatic ovarian cancer showed that p53 hotspot mutations (G245S and Y220C) cause infiltration of mutation-reactive T cells into ovarian cancer metastases (Deniger et al., 2018). A subsequent analysis of 140 patients with multiple types of epithelium confirmed this observation (Malekzadeh et al., 2019). p53 neoantigen-specific HLA-restricted elements and TCRs were found in thirty percent of patients carrying p53 hotspot mutations. And TIL and TCR genetically engineered T cells recognize tumor cell lines that endogenously express these p53 neoantigens. These results highlight the potential of p53 mutations as targets for T cell immunization and gene therapy. Furthermore, the increased levels of p53 protein associated with its mutation are associated with the production of anti-p53 autoantibodies, reinforcing the potential role of p53 in regulating tumor antigenicity (Couch et al., 2007; Garziera et al., 2015). Although mutant p53 has shown promise in the field of immunotherapy, induction of a specific anti-tumor response can trigger immune evasion in some cases. Recent studies have demonstrated the use of a broad-acting vaccine produced by a dendritic cell/tumor cell fusion that can potentially prevent adaptive immune evasion (Humar et al., 2014).

### p53 and Immune Checkpoint Inhibitor Therapy

Although significant advances have been made in antitumour immune checkpoint inhibitor (ICI) therapy, only a minority of cancer patients respond well to immune checkpoint inhibitors (Fares et al., 2019). An effective adaptive immune response requires efficient entry of fully activated cytotoxic T cells into the tumor environment and sufficient tumor-associated antigens that are presented on major histocompatibility complex (MHC) by antigen-presenting cell (APC) (Brown et al., 2018). However, many neoantigen-rich tumors fail to produce a positive immune response in many cancer patients (Stronen et al., 2016). Therefore, amplification of neoantigen libraries remains a promising direction for improving ICI treatment. In recent years, the concept of immunogenic cell death (ICD) has emerged, whereby dying cells stimulate an immune response to antigens released especially from dead cancer cells (Kroemer et al., 2013). Immunochemotherapy has been shown to sensitize tumors to anti-PD1 antibody therapy using clinically relevant mouse models of checkpoint inhibitor resistance (Pfirschke et al., 2016). In addition, Ad-p53 (p53 adenovirus) tumor suppressor immunogene therapy significantly reverse anti-PD-1 resistance in mouse models (Sobol et al., 2017). All these results suggest that chemotherapy-induced p53-dependent apoptosis facilitates the induction of immunogenesis. Indeed, nutlin-3-induced local p53 activation could alter the immune landscape of TME and enhance antitumour immunity by inducing ICD (Guo et al., 2017).

## Conclusion

As a tumor suppressor, the cell-autonomous function of p53 in suppressing malignant tumors has been extensively studied. More recently, growing evidence suggest a potential link between p53 and immune function, and dysfunction of p53 is also associated with inflammatory diseases. Dysfunction of p53 in tumors is shown to regulate not only immune recognition but also affect the stromal compartment, which plays an important role in controlling tumor progression. Thus, as a “guardian of genomic integrity,” p53 also functions in response to homeostatic stress, including innate and adaptive immunity as described above. There are still many uncharacterized issues that presumably have a broad impact on immunity and inflammation, which may ultimately lead to tumor development. For instance, how exactly p53 dysregulation affects the immune response to various external or internal stimuli, and what is the role of p53 in immune cell development. Moreover, depletion or mutation of p53 is likely to reprogram the microenvironment, especially the extracellular components in tumors, but the molecular regulatory mechanisms involved remain still largely unknown. p53 mutations can promote tumor cell metastasis. How the immune regulation and response are changed during this process, and in particular which immune cells’ functions are altered. In addition, the role of p53 in the remote regulation and communication between different tissues or organs will also be a highly anticipated research direction. There is no doubt that, understanding these issues will significantly improve our knowledge of both biologic and pathologic functions of p53, allowing for the development of targeted therapeutic approaches in the future.

## Author Contributions

DS and PJ conceived the manuscript. DS reviewed the literature, drafted the manuscript, and drew the figures. PJ revised the manuscript. Both authors read and approved the final manuscript.

## Conflict of Interest

The authors declare that the research was conducted in the absence of any commercial or financial relationships that could be construed as a potential conflict of interest.

## Publisher’s Note

All claims expressed in this article are solely those of the authors and do not necessarily represent those of their affiliated organizations, or those of the publisher, the editors and the reviewers. Any product that may be evaluated in this article, or claim that may be made by its manufacturer, is not guaranteed or endorsed by the publisher.
